# Efficient Catalyst One-Pot Synthesis of 7-(Aryl)-10,10-dimethyl-10,11-dihydrochromeno[4,3-b]chromene-6,8(7H,9H)-dione Derivatives Complemented by Antibacterial Activity

**DOI:** 10.1155/2016/5891703

**Published:** 2016-07-31

**Authors:** Yasameen K. Al-Majedy, Ahmed A. Al-Amiery, Abdul Amir H. Kadhum, Abu Bakar Mohamad

**Affiliations:** ^1^Department of Chemical and Process Engineering, Universiti Kebangsaan Malaysia (UKM), 43000 Bangi, Selangor, Malaysia; ^2^Environmental Research Center, University of Technology (UOT), Baghdad 10001, Iraq; ^3^Fuel Cell Institute, Universiti Kebangsaan Malaysia (UKM), 43000 Bangi, Selangor, Malaysia

## Abstract

The problem of bacteria resistance to many known agents has inspired scientists and researchers to discover novel efficient antibacterial drugs. Three rapid, clean, and highly efficient methods were developed for one-pot synthesis of 7-(aryl)-10,10-dimethyl-10,11-dihydrochromeno[4,3-b]chromene-6,8(7H,9H)-dione derivatives. Three components are condensed in the synthesis, 4-hydroxycoumarin, 5,5-dimethyl-1,3-cyclohexanedione, and aromatic aldehydes, using tetrabutylammonium bromide (TBAB), diammonium hydrogen phosphate (DAHP), or ferric chloride (FeCl_3_), respectively. Each method has different reaction mechanisms according to the catalyst. The present methods have advantages, including one-pot synthesis, excellent yields, short reaction times, and easy isolation of product. All catalysts utilized in our study could be reused several times without losing their catalytic efficiency. All synthesized compounds were fully characterized and evaluated for their antibacterial activity.

## 1. Introduction

Treatment approaches for bacterial infections remain a challenging therapeutic problem. Despite many antibiotics and chemotherapeutics available, the emergence of old and new antibiotic resistant bacterial strains in recent decades has increased the need for new classes of antibacterial agents [1]. Coumarins are a member of significant organic compounds due to their broad medicinal applications, like antimicrobials, antitumors, anticoagulants, antithrombotic, antihuman immunodeficiency virus, and scavenger uses [2–6]. Moreover, coumarin derivatives are able to be used as urease-inhibitors [7] and corrosion inhibitors [8–11]. The impact of different substituents on coumarins as antioxidants has been profoundly examined by scientists, who discovered the number and nature of the hydroxy; alcoxy or alkyl substituents as electron-giving gatherings are the most vital elements in charge of modifying the cancer prevention agent activities of coumarins. In other words, coumarins have great cancer prevention agent properties according to nature and number of substituted hydroxy groups [12]. To extend our studies on the design and synthesis of new compounds [13–20], we describe simple and efficient methods for synthesizing new coumarin derivatives using different aromatic aldehydes, 4-hydroxycoumarin, and 5,5-dimethyl-1,3-cyclohexanedione in three methods. Each method has a different catalyst, namely, tetrabutylammonium bromide (TBAB), diammonium hydrogen phosphate (DAHP), or FeCl_3_. All catalysts are recyclable and have reproducible results without any loss of its activity. All synthesized compounds were evaluated for their antibacterial and antioxidant activity.

## 2. Results and Discussion

### 2.1. Chemistry

**Figure 1:**
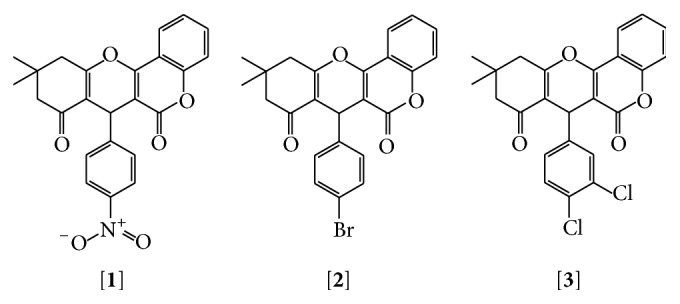
Reaction sequences of the synthesized compounds.

The reactions for the synthesis of the new compounds, namely, 7-(4-nitrophenyl)-10,10-dimethyl-10,11-dihydrochromeno[4,3-b]chromene-6,8(7H,9H)-dione [**1**], 7-(4-bromophenyl)-10,10-dimethyl-10,11-dihydrochromeno[4,3-b]chromene-6,8(7H,9H)-dione [**2**], and 7-(3,4-dichlorophenyl)-10,10-dimethyl-10,11-dihydrochromeno[4,3-b]chromene-6,8(7H,9H)-dione [**3**], were successfully synthesized and completed under conventional and novel methods and the reaction sequence for the synthesis of the novel compounds is shown in Figure 1, starting from 4-hydroxycoumarin. Many researchers synthesized coumarin derivatives from 4-hydroxycoumarin [21–24].

All compounds were synthesized by the reaction of 4-hydroxycoumarin, 5,5-dimethyl-1,3-cyclohexanedione, and aromatic aldehydes (p-nitrobenzaldehyde, p-bromobenzaldehyde, and 3,4-dichlorobenzaldehyde) with various catalysts (tetrabutylammonium bromide (TBAB) as a catalyst for synthesis of novel compounds: method A using diammonium hydrogen phosphate (DAHP) as catalyst for synthesis of novel compounds; method B and a novel method using of FeCl_3_ as a catalyst for the synthesis of the target compounds; method C). The FT-IR spectrum for 7-(4-nitrophenyl)-10,10-dimethyl-10,11-dihydrochromeno[4,3-b]chromene-6,8(7H,9H)-dione [**1**] showed an absorption band at 1687.1 cm^−1^ from the stretching of the carbonyl (-C=O) and at 1344 cm^−1^ and 1274.4 cm^−1^ for C-O-C symmetric and asymmetric stretching, respectively. The ^1^H-NMR spectrum showed a singlet at *δ* 1.3 ppm that was due to the methylene protons (2H of CH) and a triplet at *δ* 1.42 ppm that was due to methylene protons (2H of CH_2_). The ^13^C-NMR spectrum showed significant signals at 205.0 for the carbon-carbonyl group and 168.38 for the carbon attached to the nitro group. The FT-IR spectrum for 7-(4-bromophenyl)-10,10-dimethyl-10,11-dihydrochromeno[4,3-b]chromene-6,8(7H,9H)-dione [**2**] showed an absorption band at 1705.00 and 1688.4 cm^−1^ due to the stretching of the carbonyls (-C=O) and at 1485.8 cm^−1^ and 1267.2 cm^−1^ for C-O-C symmetric and asymmetric stretching, respectively. The ^1^H-NMR spectrum showed a singlet at *δ* 1.21 ppm that was due to the methylene protons (2H of CH) and a triplet at *δ* 1.38 ppm that was due to methylene protons (2H of CH_2_). The ^13^C-NMR spectrum showed significant signals at 204.97 for the carbon-carbonyl group and 165.84 for the carbon attached to the bromide atom. The FT-IR spectrum for 7-(3,4-dichlorophenyl)-10,10-dimethyl-10,11-dihydrochromeno[4,3-b]chromene-6,8(7H,9H)-dione [**3**] showed an absorption band at 1661.8 cm^−1^ that was due to the stretching of the carbonyl (-C=O) and at 1468.8 cm^−1^ and 1263.3 cm^−1^ for C-O-C symmetric and asymmetric stretching, respectively. The ^1^H-NMR spectrum showed a singlet at *δ* 1.12 ppm due to the methylene protons (2H of CH) and a triplet at *δ* 2.02 ppm due to the methylene protons (2H of CH_2_). The ^13^C-NMR spectrum showed significant signals at 201.1 ppm for the carbon-carbonyl group and at 194.12 and 194.90 for carbon attached to chlorides atoms.

#### 2.1.1. Postulated Mechanism

**Figure 2:**
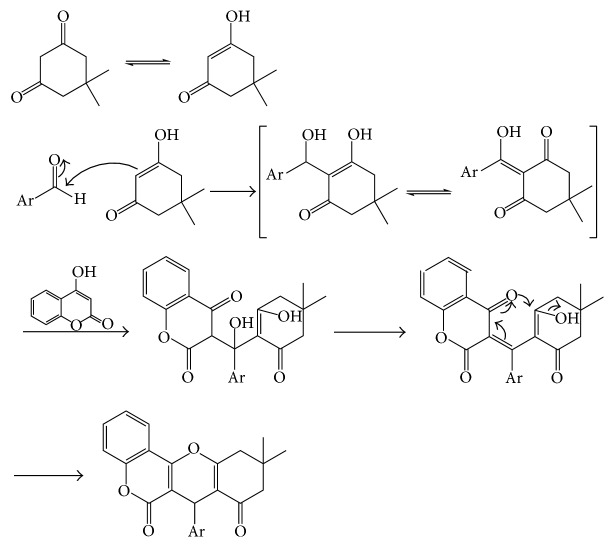
Reaction mechanism for the synthesis of compounds (**1**–**3**). Ar: [**1**]=4-nitrophenyl; [**2**]=4-bromophenyl; [**3**]=3,4-dichlorophenyl.

Figure 2 represents the suggested mechanism for the synthesis of the novel compounds by the reaction of 4-hydroxycoumarin with a dioxo-compound, namely, 5,5-dimethyl-1,3-cyclohexanedione, in presence of various catalysts.

### 2.2. Antibacterial Activity

**Figure 3:**
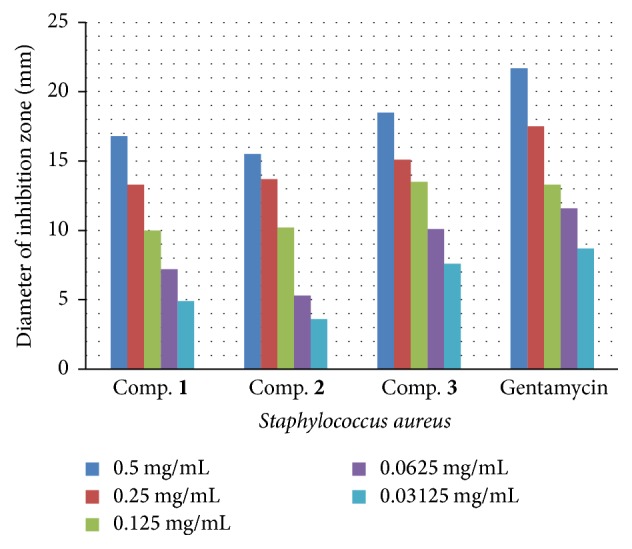
The effect of synthesized compounds (**1**–**3**) on* Staphylococcus aureus*.

**Figure 4 fig4:**
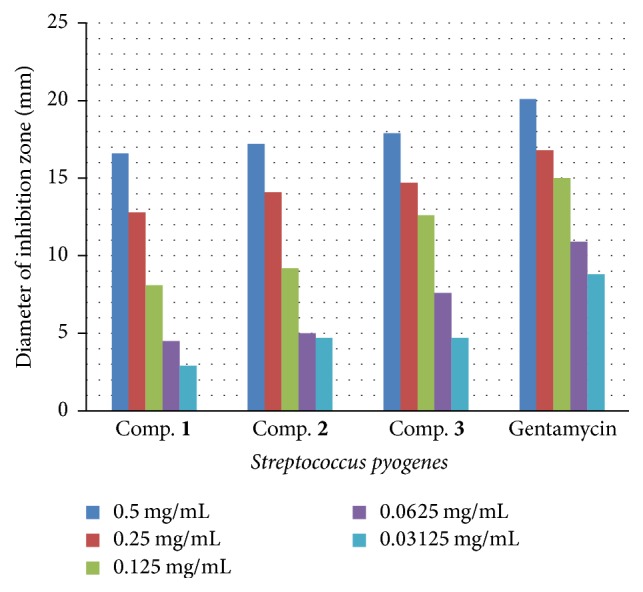
The effect of synthesized compounds (**1**–**3**) on* Streptococcus pyogenes*.

**Figure 5 fig5:**
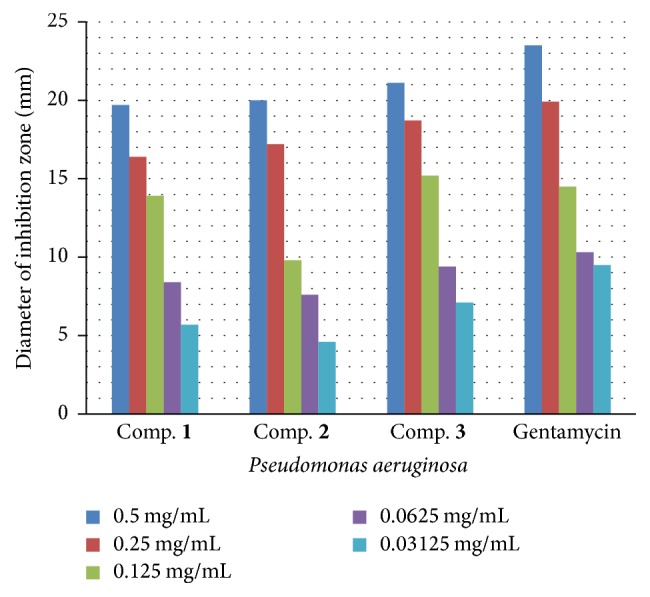
The effect of synthesized compounds (**1**–**3**) on* Pseudomonas aeruginosa*.

**Figure 6:**
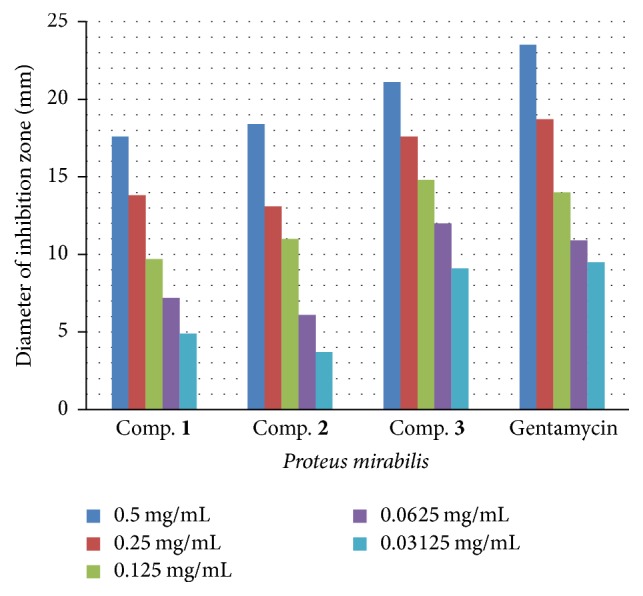
The effect of synthesized compounds (**1**–**3**) on* Proteus mirabilis*.

The antibacterial activities data demonstrate that the newly synthesized compounds (**1**–**3**) exhibit antibacterial action and that (**1**–**3**) they display additional restraint effects beyond the parent 4-hydroxycoumarin. The raised activity of the metal chelates can be explained using the fundamentals of resonance theory. Delocalization of pi-electrons above the entire synthesized molecule area electrons belonging to certain molecules is not attached to a particular atom or bond in that molecule. These electrons are said to be “delocalized” because they do not have a specific location (are not localized). Electrons become delocalized in order to stabilize a structure [25]. This raises the lipophilic capacity of the synthesized compounds (**1**–**3**), increasing its penetration through the lipoid-layer membrane of the bacteria. The lipophilic character of the synthesized compounds (**1**–**3**) was increased and seems to be responsible for the enhanced potent antimicrobial activity. The mechanism of the synthesized compounds (**1**–**3**) for killing bacteria may involve deactivation of different cellular enzymes, playing a vital role in diverse metabolic pathways of selected bacteria types. We suggest that the final action of the toxicant impairs the proteins in the cell, resulting in denaturation of natural cellular processes. Other factors that may also raise the activity are as follows: conductivity, solubility, and bonds lengths. As a result of the antibacterial screening study of (**1**–**3**), as shown in Figures 3 [Fig fig4] [Fig fig5]–6, several conclusions can be drawn. First, all compounds exhibited antibacterial activity toward all selected types of tested organisms. Second, the inhibition of gram-negative bacteria is more than that of gram-positive bacteria. Third, compound** 3** has more activity for all tested bacteria types compared with compounds** 2** and** 3** combined. Fourth, compound** 3** has two chloro-atoms, and they push electrons via the inductive effect; this may be the main mechanism by which compound** 3** acts against bacteria.

### 2.3. Antioxidant Activities

**Figure 7:**
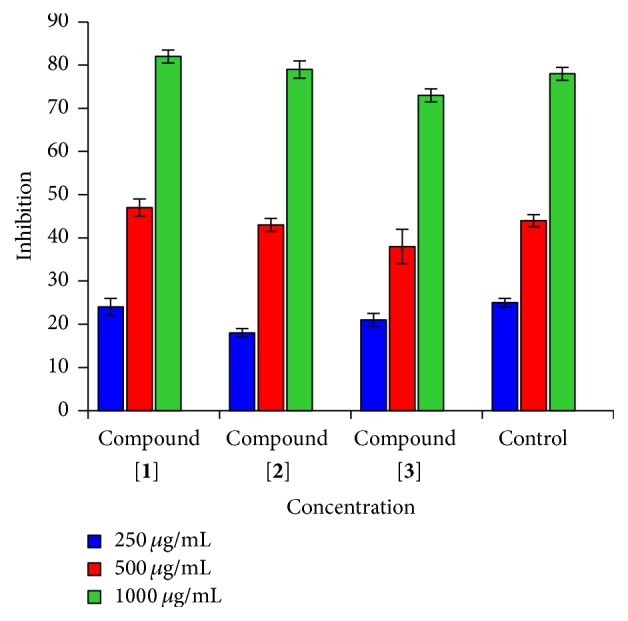
Antioxidant activity of different concentrations of the synthesized compounds [**1**,** 2** and** 3**] and Trolox in the DPPH radical scavenging assay.

1,1-Diphenyl-2-picrylhydrazyl radical (DPPH) has been a stable radical that might share an electron or hydrogen and exchange it with a steady compound. DPPH in solution shows a solid ingestion band at 517 nm because of the single electron and on the off chance that this solution was blended with reasonable reducing agent; the outcome would combine the electrons and an absence of colure. The colure has been stoichiometrically diminished as the quantity of electrons has taken up expansions, and the abatement in the absorbance could be specifically measured and contrasted and the standard (Trolox).  Figure 7 demonstrates the high DPPH radical rummaging exercises contrasted and the Trolox standard. Unmistakably all blended mixes have the capacity as antioxidant compare and Trolox.

### 2.4. Proposed Mechanism for Antioxidant of Compound [**1**]

**Figure 8:**
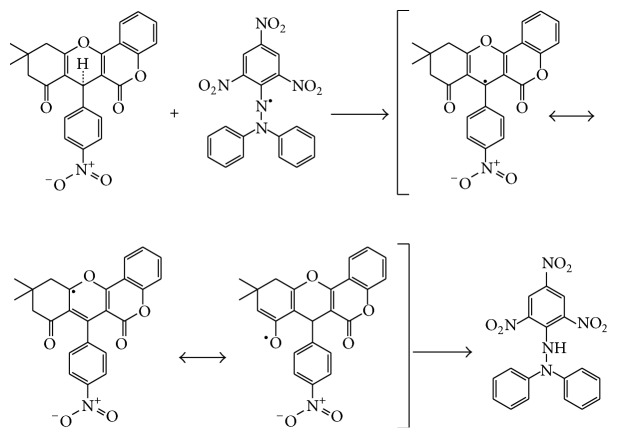
The reaction scheme between DPPH free radicals and compound [**1**].

In light of the synthetic structure, we can accept that the antioxidant impact of the compound [**1**], as appeared in Figure 8, relies on the hydrogen atoms of the benzyl group, which was under the effect of resonance and/or inductive impacts. Resonance and inductive impacts encourage the decrease of the hydrogen group, making the particle more steady. Compound [**1**] has scavenging impacts because of the stability of the intermediate radical. The elimination of a hydrogen atom from a benzyl group may happen effectively [26, 27].

## 3. Materials and Methods

### 3.1. Chemistry

All reagents were purchased from Fluka, Merck, and Sigma-Aldrich (Selangor, Malaysia), with high-grade quality and used without any purification. Reaction steps were observed by TLC (Thin Layer Chromatography) on silica gel G with solvent system benzene:ethyl acetate:methanol in percentage 40 : 30 : 30, v/v/v, respectively. Separation spots were achieved by UV lights at the Lambda max 254 and max 365 nm. All yields refer to isolated products after purification. Products were characterized by spectroscopy data. The Fourier transform infrared spectroscopy was utilizing Thermo Scientific Nicolet and the significant values are revealed in cm^−1^. Nuclear magnetic resonance (^1^H-NMR and ^13^C-NMR) spectra were recorded using an AVANCE III 600 MHz spectrometer (Bruker, Billerica, MA, USA), using DMSO as an internal standard, and the values are expressed in *δ* ppm.

#### 3.1.1. Tetrabutylammonium Bromide (TBAB) as Catalyst for Synthesis of Novel Compounds (Method A)

Reactants, namely, 4-hydroxycoumarin (0.162 g; 10 mmol) with aldehyde (5 mmol) (4-nitrobenzaldehyde or 4-bromobenzaldehyde or 3,4-dichlorobenzaldehyde), 5,5-dimethyl-1,3-cyclohexanedione (15 mmol), and tetrabutylammonium bromide (10 mol%), were refluxed. Afterwards, the mixture was cooled to room temperature. Solid mass was filtered and dried and then recrystallized from ethanol. All compounds were characterized by spectroscopic and physical data.

#### 3.1.2. Diammonium Hydrogen Phosphate (DAHP) as Catalyst for Synthesis of Novel Compounds (Method B)

Reactants in 50 mL of aqueous ethanol (50% ethanol: 50% water), including 4-hydroxycoumarin (0.162 g; 10 mmol), aldehyde (10 mmol) (4-nitrobenzaldehyde or 4-bromobenzaldehyde or 3,4-dichlorobenzaldehyde), 5,5-dimethyl-1,3-cyclohexanedione (12 mmol), and diammonium hydrogen phosphate (26.4 mg, 10 mol%), were stirred for 4 h at room temperature. After completion of the reaction, the solid mass was filtered and washed with aqueous ethanol. All compounds were characterized by spectroscopic nd physical data.

#### 3.1.3. The Novel Method Using of FeCl_3_ as a Catalyst for the Synthesis of the Target Compounds (Method C)

Reactants in 100 mL water, including 4-hydroxycoumarin (0.162 g; 10 mmol), aromatic aldehyde (4-nitrobenzaldehyde or 4-bromobenzaldehyde or 3,4-dichlorobenzaldehyde) (10 mmol), 5,5-dimethyl-1,3-cyclohexanedione (10 mmol), and FeCl_3_ (10 mol%), were refluxed for 20 h at 70°C. After completion of the reaction, the product was filtered and washed with aqueous ethanol (33% ethanol: 67% water). The filter cake recrystallized from ethanol. All compounds were characterized by spectroscopic and physical data.

7-(4-Nitrophenyl)-10,10-dimethyl-10,11-dihydrochromeno[4,3-b]chromene-6,8(7H,9H)-dione [**1**], (FeCl_3_): 35%. (TBAB) 65%. (DAHP) 45% yield; mp: 207–210°C (Reference; M.P. = 208–210°C  [28]); FT-IR: 3399.8 (C-H aromatic), 2962.5 (C-H aliphatic), 2874.8 (C-H aliphatic), 1687.1 (C=O), 1610.1 (C=C alkene), 1571.1 (C=C aromatic), 1515.7 (C=C aromatic), 1344 (C-O-C symmetric), and 1274.4 (C-O-C asymmetric), cm^−1^; ^1^H-NMR *δ*: 1.3 (s, 2H, CH_2_), 1.42 (t, 2H, CH_2_), 1.8 (t, 2H, CH_2_), 3.9 (d, 1H, CH), 7.10–7.80 (m, 4H, aromatic.), 7.4–8.1 (m, 4H, aromatic.), ^13^C-NMR; 12.99, 19.50, 23.26, 58.51, 103.22, 104.25, 113.95, 115.43, 115.93, 123.01, 123.70, 124.52, 127.90, 128.07, 128.18, 129.71, 131.84, 140.58, 145.74, 146.06, 152..22, 153.88, 163.31, 164.81, 168.38, 205.

7-(4-Bromophenyl)-10,10-dimethyl-10,11-dihydrochromeno[4,3-b]chromene-6,8(7H,9H)-dione [**2**], (FeCl_3_): 30%, (TBAB) 60% yield; (DAHP) 40% yield; mp: 133–135°C; FT-IR: 3075 (C-H aromatic), 2960.8, (C-H aliphatic), 2873.7 (C-H aliphatic), 1705 (C=O carbonyl), 1688.4 (C=O carbonyl), 1612.7, (C=C alkene), 1571.1, (C=C aromatic), 1485.8 (C-O-C symmetric), and 1267.2 (C-O-C asymmetric), cm^−1^; ^1^H-NMR *δ*: 1.21 (2H, CH_3_), 1.38 (t, 2H, CH_2_), 2.1 (t, 2H, CH_2_), 2.4 (t, 1H, CH), 7.17–7.20 (m, 4H, aromatic), 7.3–7.9 (m, 4H, aromatic) ^13^C-NMR *δ*. 13.00, 19.50, 23.56, 58.49, 103.57, 104.90, 114.15, 118.79, 122.49, 123.96, 128.16, 129.10, 130.41, 130. 47, 130.60, 131.04, 132.38, 138.48, 144.17, 152.71, 162.84, 165.84, 204.97.

7-(3,4-Dichlorophenyl)-10,10-dimethyl-10,11-dihydrochromeno[4,3-b]chromene-6,8(7H,9H)-dione [**3**], (FeCl_3_,): 30% yield; (TBAB) 60% yield; (DAHP) 45% yield; mp: 83–85°C; FT-IR: 3050 (C-H aromatic), 2961.2 (C-H aliphatic), 2873.4 (C-H aliphatic), 2149.5, 1690.7 (C=O), 1661.8(C=O), 1610.7(C=C alkene), 1571.1 (C=C aromatic), 1468.8 (C-O-C asymmetric) and 1263.3 (C-O-C symmetric), cm^−1^; ^1^H-NMR *δ*: 1.12 (s, 2H, CH_3_), 2.02 (t, 2H, CH_2_), 2.26 (t, 2H, CH_2_), 2.4 (t, 1H, CH), 7.2–7.3 (m, 4H, arom.), 7.4–7.9 (m, 4H, arom.). ^13^C-NMR *δ*. 26.26, 41.47, 46.29, 102.93, 111.43, 114.26, 120.07, 123, 123.62, 127.16, 127.37, 127.94, 130.27, 130.70, 131.05, 131.24, 149.14, 153.06, 153.10, 194.12, 194.90, 201.1.

### 3.2. Antibacterial Activities

Antibacterial activities of the novel synthesized coumarins were examined utilizing diffusion technique [29] on G+ bacteria species named (*Staphylococcus aureus* and* Streptococcus pyogenes*) and G− bacteria species named (*Pseudomonas aeruginosa* and* Proteus mirabilis*). A nutrient agar was utilized to estimate the antimicrobial activity. Results were demonstrated as inhibition zones and shown in mm. Stock solutions at a concentration of 1 mg/mL for synthesized target compounds were prepared by dissolving the target compounds in dimethyl sulfoxide, and the inhibition zone was measured (in millimeters) at 37°C after an 18 h incubation period. The concentrations of the prepared solutions were as follows: 0.5, 0.25, 0.125, 0.0625, and 0.03125 mg/mL. To clarify any effect of DMSO on biological screening, a separate study was performed using dimethyl sulfoxide (99.5% DMSO) as a control, and the result showed that there was no activity against any of the selected bacteria. For comparison, a standard antibiotic drug, gentamycin, was used as a standard for antimicrobial activity.

### 3.3. Antioxidant Activity

It is DPPH radical scavenging impact.

The DPPH radical scavenging action measure [30] was one with trifling alterations. Different concentrations of the investigated compounds [**1**,** 2** and** 3**] (250, 500, and 1000 mg/mL) were utilized. The DPPH solution was set up by dissolving 6.0 mg of this compound in 100 mL of the dissolvable (methyl alcohol). At that point, 1 mL of every concentration was added to 2 mL of the DPPH solution. At the end, the control has been set up by adding 1 mL of methyl alcohol to 2 mL of DPPH. Trolox was utilized as the standard. The mixture has been shaken vivaciously and dark incubated for 30 min. The absorbance of the solutions was spectrophotometrically investigated at 517 nm. The scavenging impacts of each compound of the DPPH radicals were evaluated utilizing

(1) $$\begin{array}{c} {\text{DPPH}}_{\text{scavenginig effect}}\;\%=A^{o}-\frac{A}{A^{o}}\times 100. \end{array}$$

### 3.4. Statistical Analysis

The results were expressed as the mean ± standard deviation, and the statistical significance of differences was determined using ANOVA (one-way analysis of variance) with SPSS 17.0 statistical software program. Significant differences were considered at *P* < 0.05, and values are presented as the mean ± SD (*n* = 3).

## 4. Conclusions

New macrocompounds, namely, 7-(4-nitrophenyl)-10,10-dimethyl-10,11-dihydrochromeno[4,3-b]chromene-6,8(7H,9H)-dione [**1**], 7-(4-bromophenyl)-10,10-dimethyl-10,11-dihydrochromeno[4,3-b]chromene-6,8(7H,9H)-dione [**2**] , and  7-(3,4-dichlorophenyl)-10,10-dimethyl-10,11-dihydrochromeno[4,3-b]chromene-6,8(7H,9H)-dione [**3**], were successfully synthesized using conventional and novel methods. The characterization of these compounds (S1, S2, and S3 Figs) was performed with different spectroscopic techniques (FT-IR, ^1^H-NMR, and ^13^C-NMR). The antibacterial activity of these compounds was determined by using gram-positive bacteria (*Staphylococcus aureus* and* Streptococcus pyogenes*) and gram-negative bacteria (*Pseudomonas aeruginosa* and* Proteus mirabilis*). The results indicated that the new compounds have higher antibacterial activity than gentamycin. The synthesized compounds had good antibacterial activity against both types of bacteria that were evaluated (gram-positive and gram-negative), and these notable antibacterial effects of the synthesized compounds confirm the basis for synthesizing new series that are derived from these synthesized compounds. The availability of these compounds will also facilitate further investigations of their pharmacological properties.
